# Supplementary material for: Optimizing Treatment Strategies for Egfr-Mutated Non-Small-Cell Lung Cancer Treated with Osimertinib: Real-World Outcomes and Insights
Source: Cancers (Basel). 2024 Oct 23;16(21):3563. doi: 10.3390/cancers16213563 (PMC11545234; doi:10.3390/cancers16213563)
Supplement: Supplementary file 1 [file cancers-16-03563-s001.zip › cancers-3234052-supplementary.pdf]

cancers

MDPI

Supplementary Materials

Table S1. Prognostic factors associated to OS—Multivariable analysis with Cox model—Osimertinib in L1.

Parameter	Classes	Hazard Ratio [IC95%]	P-value	Global *p*-value
ECOG Status (0–1) at index date	2+ vs 0–1	2.127 [1.183–3.823]	0.012	0.013
	Not available vs 0–1	0.951 [0.555–1.631]	0.855	
Metastasis site (Brain) at index date	No vs Yes	0.923 [0.570–1.494]	0.745	0.745
Metastasis site (Liver) at index date	No vs Yes	1.036 [0.561–1.913]	0.911	0.911
Metastasis site (Bone) at index date	No vs Yes	0.871 [0.549–1.380]	0.556	0.556
Age at index date	[60–65] vs <60	1.031 [0.457–2.328]	0.942	0.932
	[65–70[vs <60	1.186 [0.497–2.832]	0.701	
	≥70 vs <60	0.915 [0.513–1.632]	0.763	
Smoking status	Current smoker vs Never smoked	1.394 [0.547–3.556]	0.487	0.317
	Ex-smoker vs Never smoked	0.790 [0.462–1.352]	0.39	
	Unknown vs Never smoked	0.531 [0.232–1.218]	0.135	
Exon 19 or l858R mutation category	With L858R vs Exon19del only	1.733 [1.089–2.757]	0.02	0.02
Gender	Female vs Male	1.901 [1.061–3.407]	0.031	0.031
Testing PDL1	Positive vs Negative	1.381 [0.788–2.422]	0.26	0.525
	Others vs Negative	1.197 [0.687–2.087]	0.526	
Radiotherapy or radiosurgery at index line	No vs Yes	0.801 [0.450–1.426]	0.451	0.451
Mutation T790M	No vs Yes	0.612 [0.254–1.476]	0.274	0.274

Table S2. Prognostic factors associated to OS–Multivariable analysis with Cox model–Osimertinib in L2.

Parameter	Classes	Hazard Ratio [IC95%]	P-value	Global *p*-value
ECOG Status (0–1) at index date	2+ vs 0–1	2.248 [1.489–3.395]	<0.001	<0.001
	Not available vs 0–1	1.116 [0.779–1.598]	0.55	
Metastasis site (Brain) at index date	No vs Yes	0.934 [0.725–1.203]	0.597	0.597
Metastasis site (Liver) at index date	No vs Yes	0.666 [0.505–0.877]	0.004	0.004
Metastasis site (Bone) at index date	No vs Yes	0.774 [0.595–1.006]	0.056	0.056
Age at index date	[60–65] vs <60	0.814 [0.544–1.217]	0.315	0.047
	[65–70[vs <60	1.318 [0.855–2.032]	0.211	
	≥70 vs <60	1.291 [0.939–1.776]	0.116	
Smoking status	Current smoker vs Never smoked	1.113 [0.699–1.772]	0.651	0.947
	Ex-smoker vs Never smoked	0.959 [0.714–1.289]	0.781	
	Unknown vs Never smoked	1.020 [0.613–1.697]	0.94	
Exon 19 or l858R mutation category	With L858R vs Exon19del only	1.583 [1.234–2.031]	<0.001	<0.001
Gender	Female vs Male	0.733 [0.546–0.985]	0.039	0.039
Testing PDL1	Positive vs Negative	1.569 [1.063–2.317]	0.023	0.025
	Others vs Negative	1.032 [0.736–1.447]	0.856	
Radiotherapy or radiosurgery at index line	No vs Yes	1.159 [0.853–1.574]	0.347	0.347
BMI at index date	Underweight <18.5 vs Normal [18.5–25]	1.527 [0.726–3.210]	0.264	0.277
	Overweight/obese ≥25 vs Normal [18.5–25[	1.348 [0.876–2.073]	0.174	
	Not available vs Normal [18.5–25[	1.433 [0.953–2.155]	0.084	
Mutation T790M	No vs Yes	1.220 [0.928–1.605]	0.154	0.154
